# Feeling or thinking? User aversion to AI customer service in online education

**DOI:** 10.3389/fpsyg.2026.1956842

**Published:** 2026-10-15

**Authors:** Yuan He, Lin Huang

**Affiliations:** 1 School of Economics and Management, Dongguan University of Technology, Dongguan, China; 2 College of Management and Economics, Tianjin University, Tianjin, China; 3 School of Humanities Education and Application, Sichuan Technology and Business University, Chengdu, China; 4 Faculty of Humanities and Social Sciences, City University of Macau, Taipa, Macao SAR, China

**Keywords:** AI customer service, AI identity disclosure timing, online education, perceived intelligence, user aversion

## Abstract

**Introduction:**

AI customer service is increasingly integrated into online education, yet users may not always respond favorably to AI-assisted service interactions. Drawing on robotic role theory, this study examines how thinking and feeling AI customer service influence user aversion in online educational service encounters.

**Methods:**

Three experiments were conducted to examine the effect of AI customer service type on user aversion (Study 1, *N* = 120), the mediating role of perceived intelligence (Study 2, *N* = 120), and the moderating role of AI identity disclosure timing (Study 3, *N* = 120). Across the three between-subjects experiments, thinking AI was operationalized through analytical, information-processing, and task-oriented communication, whereas feeling AI emphasized emotional recognition and relational support.

**Results:**

The results show that feeling AI generates greater user aversion than thinking AI. Perceived intelligence mediated this effect, such that feeling AI is perceived as less intelligent, which in turn increases user aversion. Further, AI identity disclosure timing moderates the indirect effect through perceived intelligence. Early disclosure of AI identity attenuates the negative effect of feeling AI.

**Discussion:**

These findings demonstrate that the effectiveness of different AI customer service types depends on the service context and highlight the importance of aligning AI communication with users’ task-oriented service needs.

## 1 Introduction

With the rapid development of generative artificial intelligence (AI) and conversational technologies, AI customer service has become increasingly embedded in digital platforms and service encounters (Cheng et al., 2024; Ouyang et al., 2022). Online education, supported by internet and big-data technologies, has overcome many temporal and spatial constraints of traditional education and enabled more flexible forms of learning (Wang et al., 2024). It encompasses not only formal educational platforms but also market-oriented training, tutoring, and examination-preparation services (Kim et al., 2021). As AI technologies continue to develop, their applications in online education have expanded from relatively standardized programmatic support to more interactive functions, including intelligent tutoring, course consultation, automated question answering, and personalized learning assistance (Vafaei-Zadeh et al., 2025). Consequently, online education firms increasingly employ AI customer service across different customer journey touchpoints, including course consultation, enrollment guidance, learning support, and after-sales service (Davenport et al., 2020).

Despite the growing adoption of AI customer service, users do not uniformly accept AI-based service interactions. Although AI customer service can improve accessibility, efficiency, and scalability (Davenport et al., 2020), consumers may still prefer human service providers and exhibit reluctance toward AI-based interactions (Flavián and Casaló, 2021; Yao et al., 2023). This issue is particularly relevant to online education, where AI customer service may be involved in information provision, course consultation, learning assistance, and problem solving. Such interactions require AI agents to perform service roles that involve different forms of communication and assistance. Therefore, an important question is how the way in which AI enacts its service role shapes users’ responses.

One characteristic of AI customer service concerns the orientation of its interaction. Drawing on the distinction between thinking intelligence and feeling intelligence proposed by Huang and Rust (2021), this study distinguishes between thinking AI customer service and feeling AI customer service. Thinking AI customer service emphasizes analytical and task-oriented communication, such as processing user information, analyzing relevant data, comparing alternatives, and providing information-based recommendations. Feeling AI customer service emphasizes affective and relational communication, such as recognizing users’ emotional expressions, acknowledging concerns, providing reassurance and encouragement, and offering interpersonal support. This distinction concerns the primary orientation of role enactment rather than the presence or absence of information or emotional content. Both forms of AI customer service may perform the same service task, but they differ in the communication resources through which the service role is enacted.

Prior research on conversational AI has examined anthropomorphism, social cues, conversational styles, empathy, social presence, and human-like communication (Araujo, 2018; Crolic et al., 2022; Feine et al., 2019; Liu-Thompkins et al., 2022). These studies demonstrate that communication characteristics can shape consumers’ perceptions and evaluations of AI service interactions. However, the appropriateness of a particular communication orientation may depend on the service role and task performed by the AI agent. Research on human-robot service interaction suggests that service robots may enact different frontline roles and that consumers’ evaluations are shaped by the behaviors through which these roles are enacted, the suitability of robots for particular service roles, and the broader interaction context (Blaurock et al., 2022). Research on AI agents’ emotional expression indicates that the effectiveness of affective communication can vary according to service task and relationship norms, with emotional expression being more suitable for some service encounters than for others (Han et al., 2023). Thus, although prior research has established that AI communication can shape service evaluations, it remains insufficiently understood whether the appropriateness of cognitive versus affective communication depends on the specific service goal or role performed by the AI agent.

Role theory provides a basis for understanding how actors perform and evaluate roles in social interactions (Biddle, 1986; Solomon et al., 1985). In human-robot service interactions, robotic role theory further emphasizes the roles enacted by robots, the behaviors through which these roles are performed, the suitability of robots for particular service roles, and the outcomes of joint robot-consumer role play (Blaurock et al., 2022). From this perspective, AI customer service is an actor performing a service role within a particular interaction context. Different communication orientations represent different ways of enacting that role. Accordingly, examining thinking AI and feeling AI from a role perspective can help explain why an interaction style that may be effective in one service context does not necessarily produce favorable consumer responses in another.

Accordingly, this study examines how different AI customer service types influence user aversion in online education. Drawing on robotic role theory, we distinguish between thinking AI and feeling AI according to their primary modes of service role enactment and examine their differential effects on users’ responses in online educational service encounters. We further examine perceived intelligence as a psychological mechanism underlying this effect and AI identity disclosure timing as a boundary condition. By linking AI communication orientation with the service role and context in which it is enacted, this study extends research on AI customer service by highlighting the contextual appropriateness of different forms of AI service communication.

The structure of the paper is as follows. Section 2 develops the theoretical framework and reviews the relevant literature. Section 3 develops the hypotheses. Section 4 presents the pretest and stimulus development procedures and provides an overview of the research design. Sections 5–7 report studies 1–3, respectively. Section 8 concludes the paper and discusses the theoretical implications, practical implications, limitations, and directions for future research.

## 2 Theoretical framework and literature review

### 2.1 Theoretical framework

Role theory views social interaction as a process in which actors occupy and enact roles within particular social contexts (Biddle, 1986; Solomon et al., 1985). A role is associated with a set of behaviors that define how an actor participates in an interaction, while role enactment refers to the behaviors through which an actor performs that role. Thus, role-related outcomes depend not only on who performs a role, but also on how the role is enacted and whether the enacted behavior is suitable for the particular interaction context (Solomon et al., 1985). In service encounters, these role-related processes are particularly important because service providers and consumers jointly participate in the interaction and respond to each other’s enacted behaviors.

The development of robotic role theory extends this perspective to human-robot service interactions by emphasizing the interactive nature of service encounters and the different ways in which robots perform frontline service roles (Blaurock et al., 2022). Several role-related dimensions of human-robot service interaction were identified, including the robot role interface, robot role enactment, robot role suitability, consumer role behavior, role performance outcomes, and role play context. Among these dimensions, robot role enactment concerns how robots perform their service roles through observable behaviors, whereas robot role suitability concerns the extent to which a robot is considered appropriate for a particular service role. These dimensions highlight that consumer responses to robotic service depend on the behaviors through which the service role is performed and the context in which that role is enacted.

This perspective is particularly relevant to AI customer service because conversational AI increasingly performs frontline roles that were traditionally undertaken by human service employees (Schepers et al., 2022). Although the underlying service task may remain similar, AI agents can enact these roles through different interaction orientations. Research on AI and service robots suggests that consumers use observable characteristics of AI agents to form inferences about their underlying capabilities and evaluate their suitability as service providers (Huang and Rust, 2021; Blaurock et al., 2022). Accordingly, differences in AI role enactment may influence users’ perceptions of the agent’s intelligence. Perceived intelligence therefore represents a psychological mechanism through which users translate observable differences in AI role enactment into evaluations of the AI service agent (Balakrishnan et al., 2022). In AI-mediated service encounters, information concerning the identity of the service agent forms part of this interaction context and may influence how users interpret the agent’s behavior. Prior research shows that consumer responses to AI identity disclosure vary according to characteristics of the service encounter and the conditions under which AI identity information is introduced (Carney et al., 2026; Li et al., 2026). AI identity disclosure timing therefore represents a contextual condition that may shape users’ evaluations of different forms of AI role enactment.

Accordingly, drawing on role theory and robotic role theory, this study develops a framework in which AI customer service type represents differences in service role enactment, perceived intelligence captures users’ psychological evaluation of the AI agent’s capability, user aversion represents a role performance outcome, and AI identity disclosure timing defines a contextual condition that shapes users’ responses to different forms of role enactment.

### 2.2 Literature review

#### 2.2.1 AI customer service type

AI customer service refers to AI-enabled service agents that interact with customers to provide information, recommendations, assistance, and problem-solving support (Ostrom et al., 2018). With the increasing use of conversational AI in frontline service, research has examined how characteristics of AI-mediated communication shape consumers’ evaluations of service interactions. Relevant research has considered anthropomorphic cues, conversational styles, social presence, empathy, and emotional communication, demonstrating that the manner in which AI agents communicate can influence consumers’ perceptions and responses (Araujo, 2018; Crolic et al., 2022).

Drawing on the distinction between thinking intelligence and feeling intelligence proposed by (Huang and Rust, 2021), this study distinguishes between thinking AI customer service and feeling AI customer service. Thinking AI customer service emphasizes analytical reasoning, information processing, objective comparison, and task-related recommendations. Feeling AI customer service emphasizes emotional recognition, affective acknowledgment, reassurance, encouragement, and relational support. The distinction therefore concerns the dominant intelligence orientation through which the AI agent performs its service role.

Thinking AI and feeling AI should be distinguished from several related constructs in the conversational AI literature. Anthropomorphism refers to the attribution of human characteristics to nonhuman entities, while social presence concerns the perceived presence and salience of a social interaction partner (Feine et al., 2019). Empathy represents a particular capacity or behavior involving the recognition and response to others’ emotional states (Liu-Thompkins et al., 2022). Feeling-oriented communication may involve empathy and social cues, while thinking-oriented communication may also incorporate human-like or social features. Thus, the distinction between thinking and feeling AI does not concern the presence or absence of particular communication cues, but the primary intelligence orientation reflected in the AI agent’s service communication.

Accordingly, prior research establishes that AI agents can enact different service roles (Cheng et al., 2021; Han et al., 2023). However, limited research has examined whether the appropriateness of cognitive versus affective AI communication varies across service goals and how such differences in role enactment influence users’ responses to AI customer service.

#### 2.2.2 User aversion

User aversion refers to an unfavorable psychological and evaluative response toward interacting with an AI system (Filiz et al., 2021). In the context of AI customer service, this construct captures users’ negative responses to the AI-mediated service interaction itself, rather than their reluctance to rely on a particular algorithmic recommendation or decision. User aversion is related to, but conceptually distinct from, the broader literature on algorithmic aversion and AI-related resistance. Algorithm aversion primarily concerns individuals’ reluctance to rely on algorithmic advice or recommendations, even when algorithms perform effectively (Dietvorst et al., 2015). Related research further indicates that consumers may hold negative perceptions of AI when they believe that AI systems lack uniquely human capabilities or characteristics (Longoni and Cian, 2022).

Existing research has identified several antecedents of negative responses toward AI. At the individual level, demographic characteristics and individual differences can shape attitudes toward AI technologies (Cheng et al., 2024). At the task level, task uncertainty, subjectivity, and perceived risk influence individuals’ willingness to rely on AI-based recommendations (Song and Lin, 2024). At the technology level, concerns regarding algorithmic decision making, transparency, and human control can also contribute to resistance toward AI systems (Castelo et al., 2019). These findings indicate that negative responses to AI can arise from characteristics of users, tasks, and technological systems rather than from AI technology alone.

Although algorithm aversion and AI-related resistance provide an important basis for understanding negative responses to AI, user aversion concerns an unfavorable psychological and evaluative response to the AI-mediated service interaction itself, rather than only reluctance to rely on algorithmic advice. This distinction is particularly relevant in AI customer service, where users evaluate not only the recommendations provided by the system but also the interaction with the AI service agent. Accordingly, user aversion captures users’ negative responses toward the AI customer service interaction at the interaction level.

#### 2.2.3 Perceived intelligence

Perceived intelligence refers to users’ subjective evaluation of an AI system’s intelligence and capability during interaction. Rather than referring to the system’s objective computational performance, perceived intelligence concerns how capable, intelligent, and autonomous the AI system appears to users (Wang and Liu, 2026). Balakrishnan et al. (2022) conceptualized perceived intelligence in terms of an intelligent system’s ability to understand users’ needs and instructions, process relevant information, communicate clearly, and operate with a degree of autonomy. This conceptualization is consistent with recent human-AI interaction research that defines perceived AI intelligence as users’ subjective appraisal of how smart, capable, and autonomous an AI system appears to be.

Perceived intelligence has been examined as an important user perception in human-AI interaction. Because users cannot directly observe the underlying computational capabilities of an AI system, their evaluations of AI intelligence are formed partly from observable system behaviors and interaction outcomes. Research has associated perceived intelligence with users’ trust, reliance, technology acceptance, and continued use of AI systems (Balakrishnan and Dwivedi, 2021; Vafaei-Zadeh et al., 2025). In AI-supported work and learning environments, perceived intelligence has also been associated with decision-making, collaboration, and learning-related outcomes (Dellermann et al., 2019; Seeber et al., 2020).

#### 2.2.4 AI identity disclosure timing

Existing research has examined AI identity disclosure in service interactions by investigating whether users are informed that a service agent is AI-based and how such information influences subsequent responses to the interaction (Atefi et al., 2020; Huang and Dootson, 2022; Luo et al., 2021). One stream compares interactions with and without AI disclosure, focusing on whether knowledge of AI identity influences consumers’ evaluations and behavioral responses (Atefi et al., 2020). Another stream considers the content and specificity of disclosure. Carney et al. (2026) distinguish between general and specific AI disclosures and show that disclosure information can influence consumer engagement depending on the characteristics of the consumption context. Prior research has also shown that information concerning AI involvement can alter consumers’ perceptions of accuracy, trustworthiness, and legitimacy (Longoni and Cian, 2022; Sheehan et al., 2020). Research further extends the scope of disclosure by examining information about human involvement in hybrid service agents, showing that disclosure of human involvement can alter customers’ communication behavior during service interactions (Gnewuch et al., 2024). Accordingly, disclosure concerns not only whether information about AI involvement is provided but also what information is communicated to users.

A further distinction concerns when AI identity information is disclosed. AI identity disclosure timing refers to the point in the service interaction at which users receive information about the AI-based nature of the service agent. Early disclosure provides such information before or at the beginning of the interaction, whereas late disclosure provides the information after users have already experienced the service interaction (Huang and Dootson, 2022). Research suggests that the effects of disclosure timing can vary across service contexts. Yu et al. (2024), for example, show that pre-dialogue disclosure is more satisfying for high-contact services, whereas post-dialogue disclosure is more appropriate for low-contact services. Similarly, Mozafari et al. (2022) demonstrate that the effects of chatbot identity disclosure vary with service criticality and service outcomes. Li et al. (2025) further show that prior disclosure of chatbot identity influences consumers’ tolerance of subsequent service failures. These findings suggest that the consequences of AI identity disclosure depend partly on when identity information is encountered and on the characteristics of the service interaction.

AI-related disclosure therefore represents a contextual factor through which users interpret AI-mediated service experiences. Disclosure can influence how users understand the nature of the service agent and subsequently evaluate the interaction (Carney et al., 2026). Moreover, evidence that disclosure effects vary across service contact, criticality, and service outcomes suggests that the consequences of disclosure are contingent on the service context in which identity information is introduced (Yu et al., 2024). However, limited research has examined whether disclosure timing changes users’ responses to different types of AI customer service.

## 3 Hypothesis development

### 3.1 AI customer service types and user aversion

Online education is characterized by service encounters in which users frequently seek information and assistance related to concrete learning or enrollment goals, such as course selection, learning support, and problem solving (Gebauer et al., 2021; Wu et al., 2026). In such contexts, the value of AI communication depends partly on whether its communication orientation is appropriate for the task being performed. Research on AI service agents shows that emotional expression is not uniformly beneficial. Han et al. (2023) demonstrate that positive emotional expressions by AI agents can improve service evaluations for personalized tasks but may be less appropriate for standardized tasks. The effectiveness of AI communication depends on the relationship between communication style and service-task characteristics.

Service agents enact roles through their observable behaviors, and the appropriateness of role enactment depends on the service context in which the interaction occurs (Schepers et al., 2022; Solomon et al., 1985). In online education, customer service interactions are often directed toward concrete informational and problem-solving objectives. Communication that emphasizes analytical processing, comparison, and task-related recommendations is therefore closely connected to these service functions. By contrast, affective and relational communication places greater emphasis on emotional acknowledgment, reassurance, and interpersonal support. Although such communication can be valuable in service encounters involving relational or personalized goals (Blümel et al., 2024), its relevance may be lower when users seek primarily task-related educational assistance.

Accordingly, when AI customer service is used for online educational consultation and learning support tasks, an affective communication orientation may be less appropriate than an analytical orientation. The greater emphasis on relational communication may make the interaction less consistent with the functional requirements of the service encounter and consequently generate a more unfavorable response toward the AI agent.

*H1*. In online education service encounters, feeling AI customer service generates greater user aversion than thinking AI customer service.

### 3.2 The mediating role of perceived intelligence

Perceived intelligence captures users’ judgments of an AI agent’s ability to understand requests, process information, and provide appropriate responses or solutions (Akter et al., 2019). Users therefore have reason to evaluate whether an AI service agent possesses sufficient cognitive capability to perform these tasks effectively. The communication orientation of an AI agent provides users with observable cues for forming such judgments. Thinking-oriented communication makes information processing and problem-solving more salient by emphasizing analytical reasoning, comparison, and task-related recommendations. These communication characteristics provide relatively direct cues regarding the agent’s cognitive capabilities. Feeling-oriented communication, in contrast, places greater emphasis on emotional acknowledgment, reassurance, encouragement, and relational support. Although such communication may convey interpersonal responsiveness, it provides fewer cues concerning the agent’s ability to process information and solve task-related problems. This distinction is particularly consequential in online educational service encounters, where cognitive assistance constitutes an important aspect of the service task. Consistently, research on AI service interactions suggests that the appropriateness and effectiveness of emotional expression depend on the type of service task being performed (Han et al., 2023).

When users perceive an AI agent as capable of understanding their needs and providing effective solutions, they are more likely to regard the agent as competent and useful, whereas lower perceived intelligence can undermine users’ evaluations of the AI interaction (Balakrishnan et al., 2022; Vafaei-Zadeh et al., 2025). In a task-oriented online education service encounter, therefore, the relatively weaker cognitive cues provided by feeling-oriented communication may reduce perceived intelligence, which can subsequently contribute to greater user aversion. Accordingly, the following hypothesis is proposed:

*H2*. In online education service encounters, perceived intelligence mediates the relationship between AI customer service type and user aversion, such that feeling AI customer service reduces perceived intelligence, which in turn increases user aversion.

### 3.3 The moderating role of AI identity disclosure timing

Prior research suggests that the effects of AI identity disclosure depend on the characteristics of the service interaction (Kim et al., 2025; Li et al., 2026). Luo et al. (2019) found that disclosing chatbot identity before the interaction reduced customers’ perceptions of chatbot knowledgeability and empathy, whereas late disclosure mitigated this negative effect. Mozafari et al. (2022) further showed that the effect of chatbot disclosure varied with service criticality and service outcomes. Cheng et al. (2021) found that chatbot disclosure interacted with perceived empathy and friendliness in shaping consumers’ trust, with the effects also varying with task complexity. These findings indicate that the consequences of AI identity disclosure depend on the characteristics of the service interaction and the communication provided by the AI agent.

Early and late disclosure provide different information conditions for evaluating the AI service. When AI identity is disclosed before the interaction, users know that they are interacting with an AI agent when they encounter its communication. When disclosure occurs after the interaction, users first experience the agent’s communication and receive information about its AI identity only afterward. Luo et al. (2019) show that this difference in disclosure timing can influence consumers’ perceptions of chatbot knowledgeability and empathy, suggesting that the timing of identity information affects how users evaluate the AI agent.

The moderating effect of disclosure timing may become more pronounced when AI agents differ in their communication orientation. Han et al. (2023) demonstrate that the effectiveness of AI emotional expression depends on the service task, with affective communication being more appropriate for personalized and relational tasks and neutral communication being more appropriate for standardized, exchange-oriented tasks. Robotic role theory likewise suggests that consumer evaluations depend on how appropriately an AI agent enacts its service role within a particular interaction context (Blaurock et al., 2022). In task-oriented educational services, the analytical and task-focused communication of thinking AI is therefore more closely aligned with the functional requirements of the service interaction, whereas the relational communication of feeling AI may be less closely aligned with these requirements. When identity is disclosed late, users experience these communication differences before receiving information about the agent’s AI identity, which may make the difference in their evaluations more pronounced.

When identity is disclosed early, users receive information about the AI agent before experiencing its communication. Prior research indicates that advance disclosure can shape subsequent evaluations of AI service agents (Luo et al., 2019). Early disclosure may therefore reduce the extent to which differences in communication orientation determine users’ evaluations of the service interaction. Accordingly, the difference in user aversion between feeling AI and thinking AI should be weaker under early disclosure than under late disclosure. Thus, this study proposes:

*H3*. In online education service encounters, AI identity disclosure timing moderates the relationship between AI customer service type and user aversion, such that the positive effect of feeling AI customer service on user aversion is weaker under early AI identity disclosure than under late AI identity disclosure.

Drawing on robotic role theory, this study develops a conceptual framework linking AI customer service type, perceived intelligence, user aversion, and AI identity disclosure timing. Specifically, AI customer service type is conceptualized as a key antecedent of user aversion, with perceived intelligence serving as the underlying mechanism. AI identity disclosure timing is conceptualized as a boundary condition that moderates both the direct and indirect effects of AI customer service type on user aversion. The conceptual model is presented in Figure 1.

**Figure 1:**
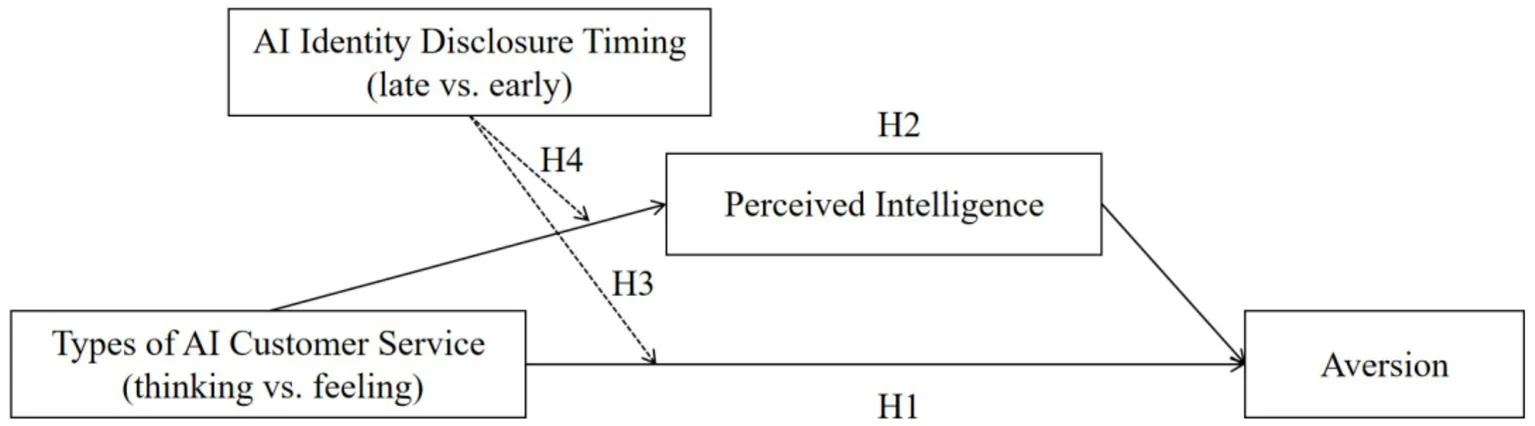
Conceptual model.

## 4 Pretest and stimulus development

Pretest was conducted to develop and validate the experimental stimuli. Following Huang and Rust (2021), five educational-service scenarios representing different stages of the online education service journey were developed. For each scenario, two matched versions of the same AI-user interaction were created, resulting in 10 interaction scripts. The two versions within each scenario were matched in service context, user profile, user request, substantive service goal, factual information, service outcome, interaction structure, approximate response length, and visual interface. The primary systematic difference concerned the orientation of AI communication. Thinking AI emphasized analytical information processing, objective comparison, diagnosis, and task-oriented recommendations, whereas feeling AI emphasized emotional acknowledgment, reassurance, encouragement, and relational support. A total of 100 participants (53.85% female; Mage = 40.37, SD = 10.23) were recruited from Credamo^1^. Each participant evaluated both the thinking and feeling AI versions of all five scenarios, with the presentation order of the two versions counterbalanced across participants. After reading each interaction, participants evaluated the perceived communication orientation of the AI agent and potential confounding characteristics, including perceived information quality (Park et al., 2024), usefulness (Davis, 1989), and competence (Longoni and Cian, 2022), using seven-point scales. Detailed measurement items and pretest results are reported in Appendix A. Based on the manipulation checks and confound checks, the three scenarios were selected as the final stimuli for Studies 1, 2, and 3, respectively. These scenarios demonstrated clear differentiation between the intended thinking and feeling orientations while maintaining comparable perceptions of service information quality, usefulness, and competence across conditions.

Three experiments were conducted to examine how AI customer service type influences user aversion in online educational service encounters and to investigate its underlying mechanism and boundary condition. Study 1 examined the effect of AI customer service type by comparing thinking and feeling AI. Study 2 replicated this effect in a study planning context and examined perceived intelligence as the underlying mechanism. Study 3 further examined the moderating role of AI identity disclosure timing using a 2 × 2 between-subjects design. Within each study, the substantive service task, user information, and service outcome were held constant across the two AI service types. The studies examined related stages of the online education service journey. Study 1 focused on pre-enrollment course consultation and selection, Study 2 on learning support and study planning, and Study 3 on vocabulary-learning support for the College English Test Band 6 (CET-6). This progression allowed the effect of AI customer service type and its underlying mechanism to be examined across related educational service contexts while maintaining a common task-oriented service setting. A summary of the three studies is provided in Appendix B. The table in Appendix B summarizes the purpose, design, educational-service touchpoint, scenario and AI task, key variables, and sample information for each study. The experimental materials for Studies 1–3 are provided in Appendices C–E. Prior to conducting ANOVA analyses, the assumptions of independence, normality, and homogeneity of variance were examined. Independence was ensured through the between-subjects experimental designs and separate recruitment of participants across studies. The distributions of dependent variables showed no substantial deviations from normality, and Levene’s tests confirmed homogeneity of variance across analyses. Participation in the survey was entirely voluntary and anonymous. Respondents were informed of the purpose of the study before completing the questionnaire, and electronic informed consent was obtained from all participants prior to participation.

## 5 Study 1: the effect of AI customer service type on user aversion

### 5.1 Method

Study 1 employed a one-factor (AI customer service type: thinking vs. feeling) between-subjects design and examined the effect of AI service orientation on user aversion in a pre-enrollment educational service context. A total of 120 participants (60% female; Mage = 30.36 years) were recruited from Credamo and randomly assigned to one of the two experimental conditions. Participants were asked to imagine that they were preparing for the civil service examination and planning to purchase an online examination-preparation course. They were uncertain about which course would be most appropriate and therefore consulted an AI customer service agent on an online education platform. The scenario represented a pre-enrollment course-consultation stage of the online education service journey, in which a prospective learner sought assistance with course selection. The AI customer service type was manipulated using the validated stimuli developed in the pretest. Participants were presented with a standardized, pre-scripted AI-user interaction through a series of screenshots. All participants within the same condition viewed exactly the same interaction content and responses. No real-time interaction with an AI system or generative AI model was involved. All participants within each condition received the same scripted interaction and responses. The complete scenario, interaction scripts, and measurement items are provided in Appendix C.

In the thinking-AI condition, the agent emphasized analytical processing, objective comparison, and information-based recommendations. It analyzed the user’s background and examination-related information to evaluate relevant options and recommend suitable courses. In the feeling-AI condition, the agent emphasized affective and relational communication by acknowledging the user’s concerns, providing reassurance and encouragement, and offering relational support while performing the same substantive service task. Thus, the manipulation concerned the primary communication orientation through which the AI agent enacted the service role, rather than differences in the substantive service outcome or information provided.

### 5.2 Measures

For the manipulation check, communication orientation (Cronbach’s *α* = 0.91) was measured using a single item on a seven-point bipolar scale ranging from affective and relational (1) to analytical and task-oriented (7). User aversion (Cronbach’s *α* = 0.95) was measured using a five-item scale adapted from Lu et al. (2019).

### 5.3 Results

*Manipulation check*. An ANOVA revealed a significant difference between the two AI customer service type (*F* (1,118) = 356.27, *p* < 0.001, *η*^2^ = 0.78). Participants in the feeling AI condition reported significantly lower scores (M_feeling_ = 2.02, SD = 0.98), indicating that the AI customer service was perceived as strongly feeling orientation, whereas participants in the thinking AI condition reported significantly higher scores (M_thinking_ = 5.38, SD = 0.97), indicating that the AI customer service was perceived as strongly thinking orientation. These results confirm that the two AI customer service type was successfully differentiated.

*User Aversion*. User aversion was treated as the dependent variable (Y), and AI customer service type (feeling vs. thinking) was specified as the independent variable (X). An ANOVA revealed a significant effect of AI customer service type on user aversion (*F* (1,118) = 56.623, *p* < 0.001, *η*^2^ = 0.32, *R*^2^ = 0.30). Participants reported higher levels of aversion toward feeling AI customer service (M_feeling_ = 5.789, SD = 1.03) than toward thinking AI customer service (M_thinking_ = 3.978, SD = 1.22). Thus, H1 was supported (see Figure 2).

**Figure 2:**
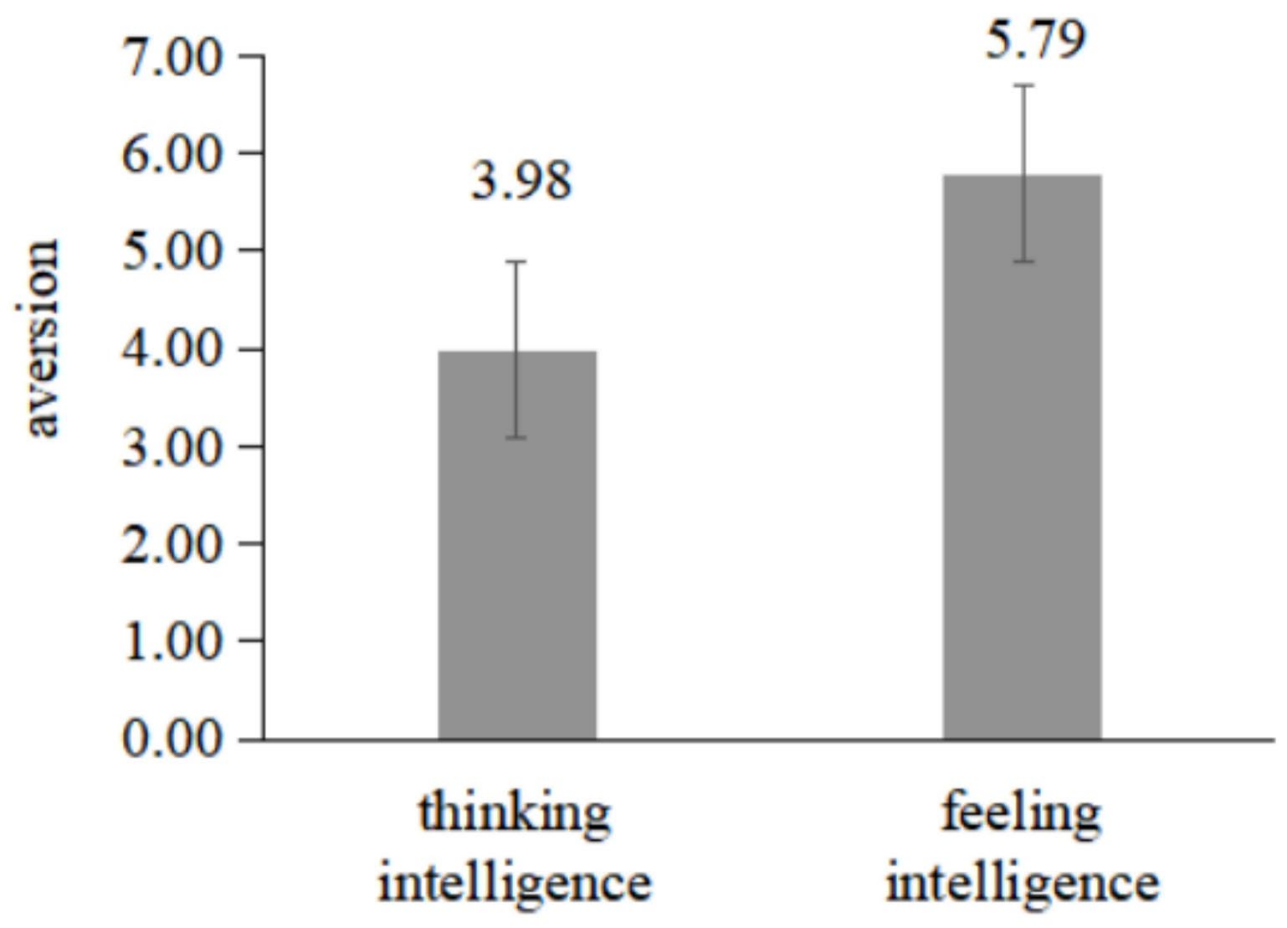
The main effect (Study 1).

## 6 Study 2: the mediating role of perceived intelligence

### 6.1 Method

Study 2 employed a one-factor (AI customer service type: thinking vs. feeling) between-subjects design, examined the mediating effect of perceived intelligence in an online programming course service context. A total of 120 participants (63.33% female; Mage = 30.38 years) were recruited from Credamo and randomly assigned to one of the two experimental conditions. Participants were asked to imagine that they had recently started learning programming through an online education platform and were completing programming exercises with the assistance of an AI learning assistant. They were beginners seeking assistance in identifying their learning difficulties and developing an appropriate learning approach. The scenario represented an online learning-support stage. The AI customer service type was manipulated using the validated stimuli developed in the pretest. The complete scenario, interaction scripts, and measurement items are provided in Appendix D.

### 6.2 Measures

Communication orientation (Cronbach’s *α* = 0.95) and user aversion (Cronbach’s *α* = 0.96) were measured using the same scales as in Study 1. Perceived intelligence (Cronbach’s *α* = 0.89) was measured using the five-item scale adapted from Balakrishnan et al. (2022).

### 6.3 Results

*Manipulation check.* An ANOVA revealed a significant difference between the two AI customer service types (*F* (1,118) = 850.32, *p* < 0.001, *η*^2^ = 0.88). Participants in the feeling AI condition reported significantly lower scores (M_feeling_ = 1.78, SD = 0.87), indicating that the AI customer service was perceived as strongly feeling orientation, whereas participants in the thinking AI condition reported significantly higher scores (M_thinking_ = 6.82, SD = 1.01), indicating that the AI customer service was perceived as strongly thinking orientation. These results confirm that the two AI customer service type was successfully differentiated.

*User Aversion.* To examine the main effect, user aversion was treated as the dependent variable (Y), and AI customer service type (feeling vs. thinking) was specified as the independent variable (X). An ANOVA revealed a significant effect of AI customer service type on user aversion (*F* (1,118) = 22.451, *p* < 0.001, *η*^2^ = 0.16, *R*^2^ = 0.20). Participants reported higher levels of aversion toward feeling AI customer service (M_feeling_ = 5.533, SD = 1.35) than toward thinking AI customer service (M_thinking_ = 4.544, SD = 0.89), providing support for H1.

*Mediating effect*. We conducted a mediation analysis using PROCESS Model 4 (version 4.1) with 5,000 bootstrap samples. AI customer service type (1 = feeling, 2 = thinking) was specified as the independent variable (X), perceived intelligence as the mediator (M), and user aversion as the dependent variable (Y). Results revealed a significant indirect effect of AI customer service type on user aversion through perceived intelligence (*β* = −0.736, SE = 0.21, CI = [−1.160, −0.348]), the direct effect was significant (*β* = −0.735, SE = 0.23, CI = [−1.201, −0.270]). These findings indicate partial mediation by perceived intelligence, supporting H2 (see Figure 3).

**Figure 3:**
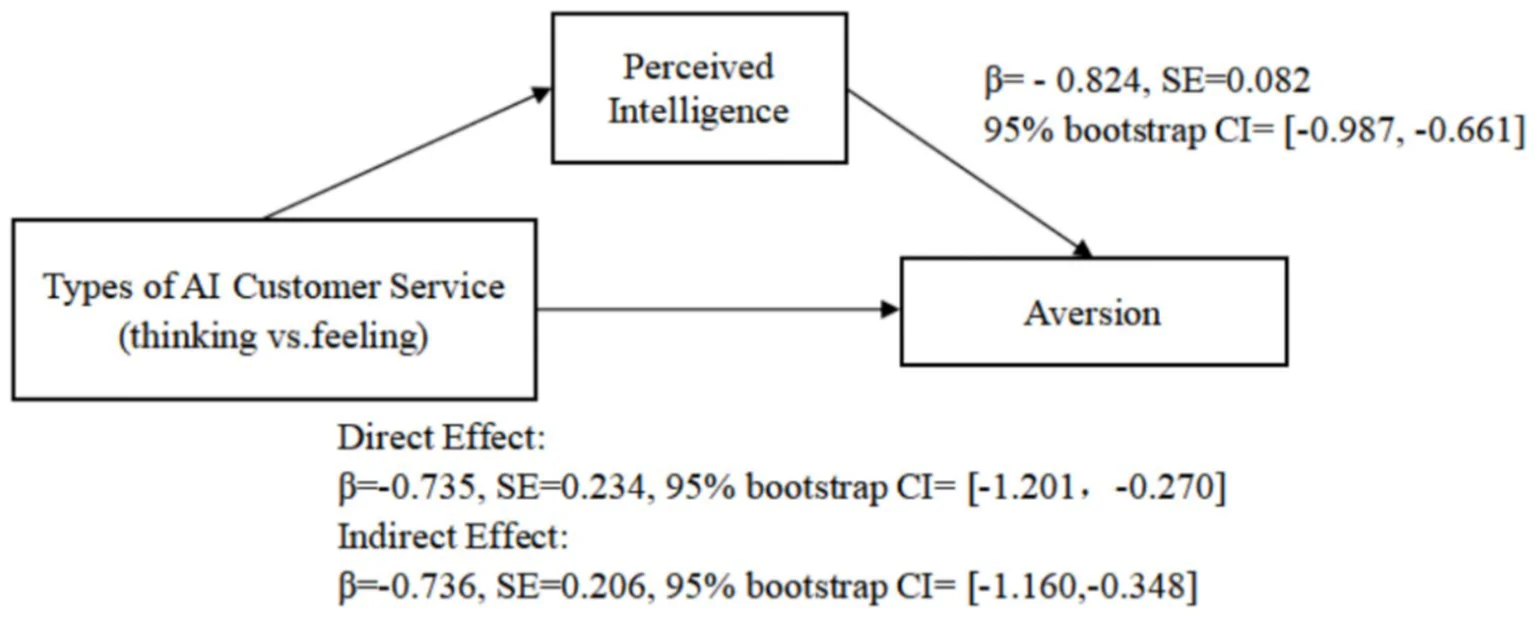
The mediating role of perceived intelligence (Study 2).

## 7 Study 3: the moderating role of AI identity disclosure timing

### 7.1 Method

Study 3 employed a 2 (AI customer service type: thinking vs. feeling) × 2 (AI identity disclosure timing: early vs. late) between-subjects design. A total of 120 participants (53.3% female; M_age_ = 30.69 years) were recruited from Credamo and randomly assigned to one of the four experimental conditions. The study simulated an educational support scenario involving preparation for the College English Test Band 6 (CET-6), a standardized English proficiency examination widely taken by university students in China. Participants were asked to imagine that they were preparing for the CET-6 and wanted to improve their English vocabulary because they perceived their current vocabulary knowledge as insufficient for the examination. They therefore consulted an online English-learning assistant integrated into a vocabulary-learning application.

The manipulation of AI service orientation followed the same response-orientation principle used in Studies 1 and 2. In the early disclosure condition, the notice was presented at the beginning of the interaction: “AI Service Notice: You are interacting with an AI learning assistant. Human customer service is available if additional assistance is required”. In the late disclosure condition, no identity notice was presented at the beginning of the interaction. Participants first completed the same AI service interaction and then received the notice at the end. Thus, both conditions received the same information regarding the AI identity of the service agent and the availability of human customer service, with the timing of disclosure differing across conditions. The complete scenario, interaction scripts, disclosure manipulations, and measurement items are provided in Appendix E.

### 7.2 Measures

Communication orientation (Cronbach’s *α* = 0.93) and user aversion (Cronbach’s *α* = 0.96) were measured using the same scales as in Study 1. Perceived intelligence (Cronbach’s *α* = 0.95) was measured as in the aforementioned studies, thereby ensuring measurement consistency and comparability across experiments.

### 7.3 Results

*Manipulation check.* The results revealed a significant difference between the two AI customer service type (*F* (1,118) = 390.22, *p* < 0.001, *η*^2^ = 0.77). Participants in the feeling AI condition reported significantly lower scores (M_feeling_ = 2.32, SD = 1.17), indicating that the AI customer service was perceived as strongly feeling orientation, whereas participants in the thinking AI condition reported significantly higher scores (M_thinking_ = 6.10, SD = 0.91), indicating that the AI customer service was perceived as strongly thinking orientation. These results confirm the effectiveness of the manipulation of AI customer service type. For AI identity disclosure timing, an ANOVA revealed that participants rated early AI identity disclosure timing significantly lower (M = 2.03, SD = 0.61) than late AI identity disclosure timing (M = 6.02, SD = 0.33, *F* (1,118) = 513.73, *p* < 0.001, *η*^2^ = 0.81), confirming the effectiveness of the AI identity disclosure timing.

*Moderating effect of AI identity disclosure timing*. Perceived intelligence was subjected to a 2 × 2 ANOVA, with AI customer service type (thinking vs. feeling) as the independent variable and AI identity disclosure timing (early vs. late) as the moderator. Results revealed a significant interaction effect between AI customer service type and disclosure timing on perceived intelligence (*F* (1,116) = 73.723, *P* < 0.001, *η*^2^ = 0.38, R^2^ = 0.35). Under the early disclosure condition, thinking AI customer service (M_thinking_ = 5.979, SD = 0.75) generated higher perceived intelligence than feeling AI customer service (M_feeling_ = 3.791, SD = 0.80; *F* (1,116) = 33.07, *p* < 0.001, *η*^2^ = 0.22). Under the late disclosure condition, thinking AI customer service (M_thinking_ = 5.313, SD = 0.65) also generated higher perceived intelligence than feeling AI customer service (M_feeling_ = 2.146, SD = 0.85; *F* (1,116) = 25.39, *p* < 0.001, *η*^2^ = 0.18) (see Figure 4).

**Figure 4:**
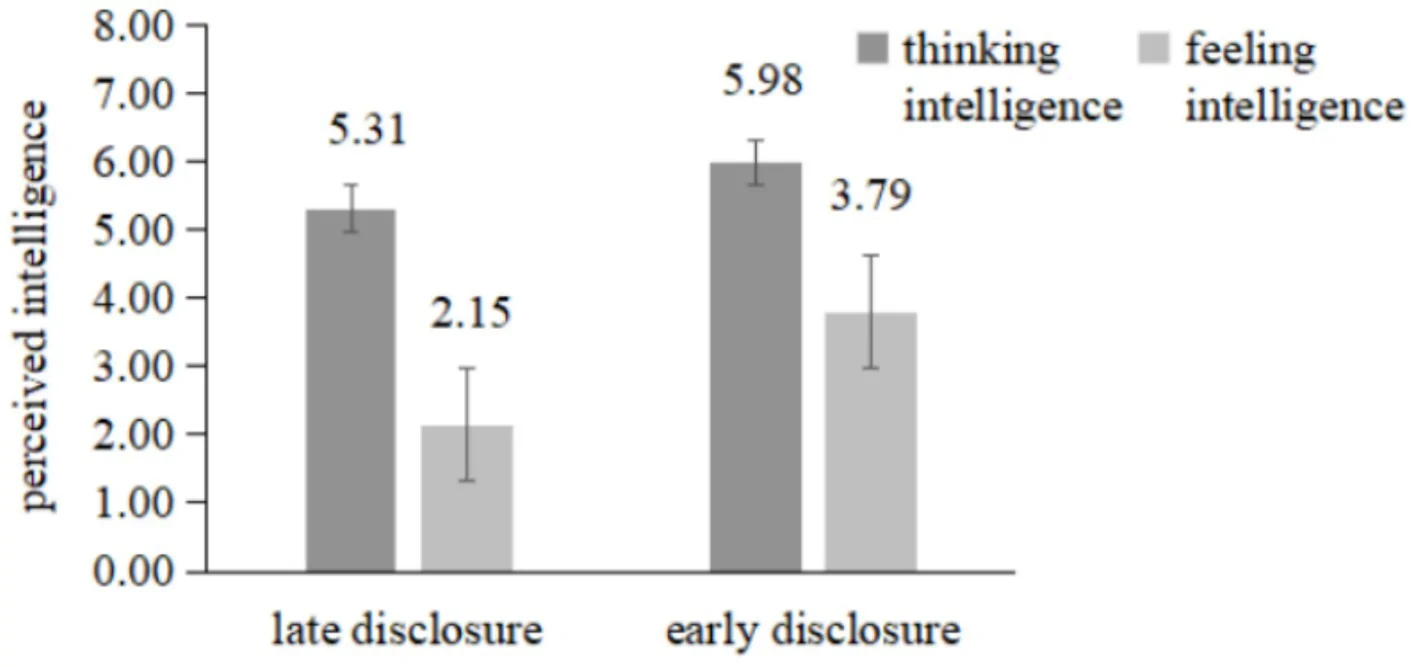
Moderating effect of Al identity disclosure timing on perceived intelligence (Study 3).

As shown in Figure 5, results revealed a significant interaction effect between AI customer service type and disclosure timing on user aversion (*F* (1,116) = 83.69, *P* < 0.001, *η*^2^ = 0.41, R^2^ = 0.24). Under the early disclosure condition, thinking AI customer service (M_thinking_ = 1.72, SD = 0.60) generated lower user aversion than feeling AI customer service (M_feeling_ = 4.09, SD = 1.06; *F* (1, 116) = 51.14, *p* < 0.001, *η*^2^ = 0.30). Under the late disclosure condition, thinking AI customer service (M_thinking_ = 2.31, SD = 0.56) also generated lower user aversion than feeling AI customer service (M_feeling_ = 5.50, SD = 0.73; *F* (1, 116) = 22.47, *p* < 0.001, *η*^2^ = 0.16). These findings provide support for H3.

**Figure 5:**
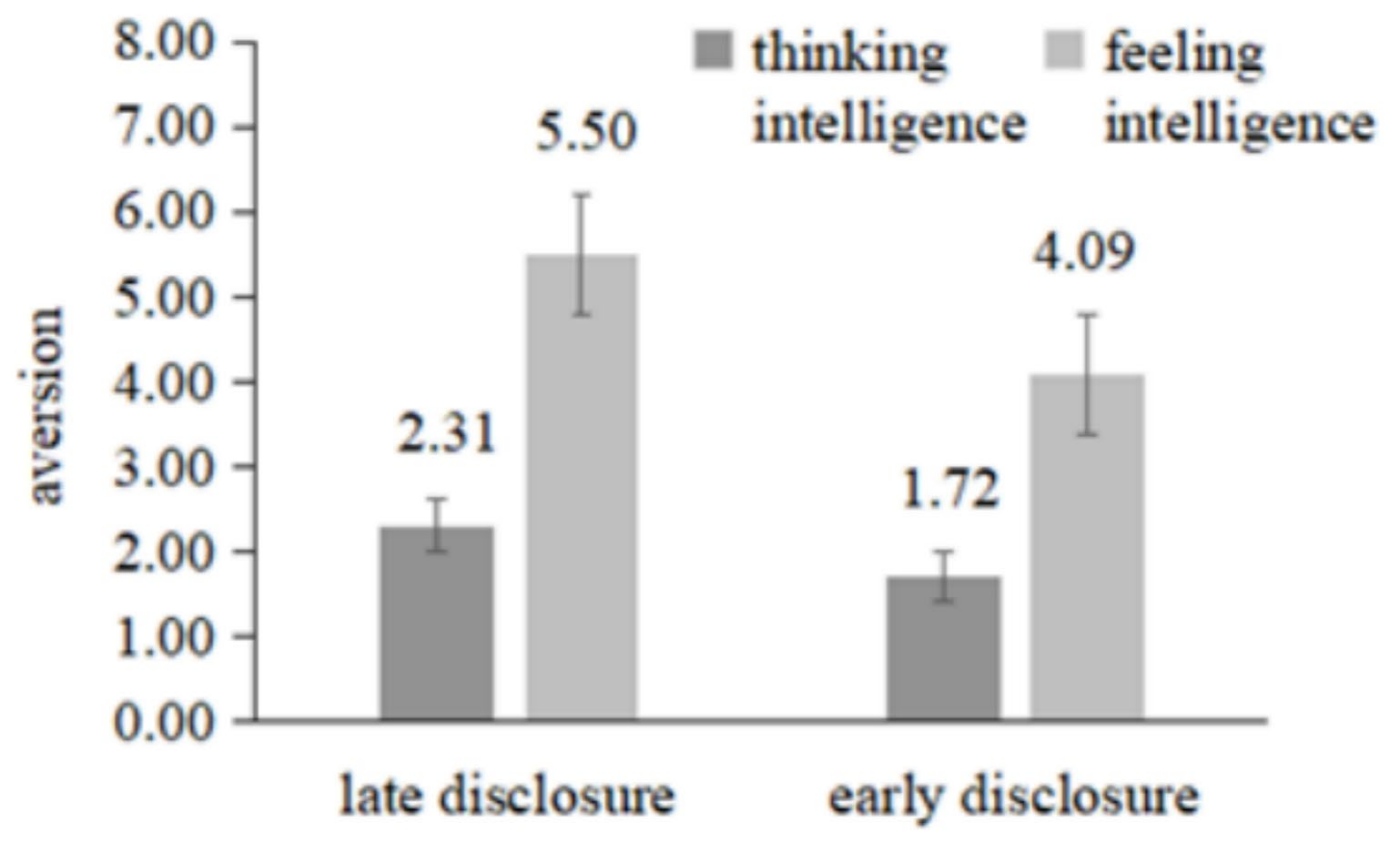
Moderating effect of Al identity disclosure timing on aversion (Study 3).

*Mediated moderation analysis.* A moderated mediation analysis was conducted using PROCESS macro (version 4.1; Model 7, 5,000 bootstrap samples), with AI customer service type (1 = feeling, 2 = thinking) was specified as the independent variable (X), AI identity disclosure timing (1 = early, 2 = late) as the moderator (W), perceived intelligence as the mediator (M), and user aversion as the dependent variable (Y). The results indicate that perceived intelligence significantly mediated the interaction effect between AI customer service type and AI identity disclosure timing on user aversion (*β* = 0.613, SE = 0.299, 95% bootstrap CI = [0.093, 1.266]). As shown in Figure 6, perceived intelligence exerts a negative mediating effect for late disclosure (*β* = −1.981, SE = 0.408, 95% bootstrap CI = [−2.826, −1.238]) and early disclosure (*β* = −1.369, SE = 0.249, 95% bootstrap CI = [−1.924, −0.927]).

**Figure 6:**
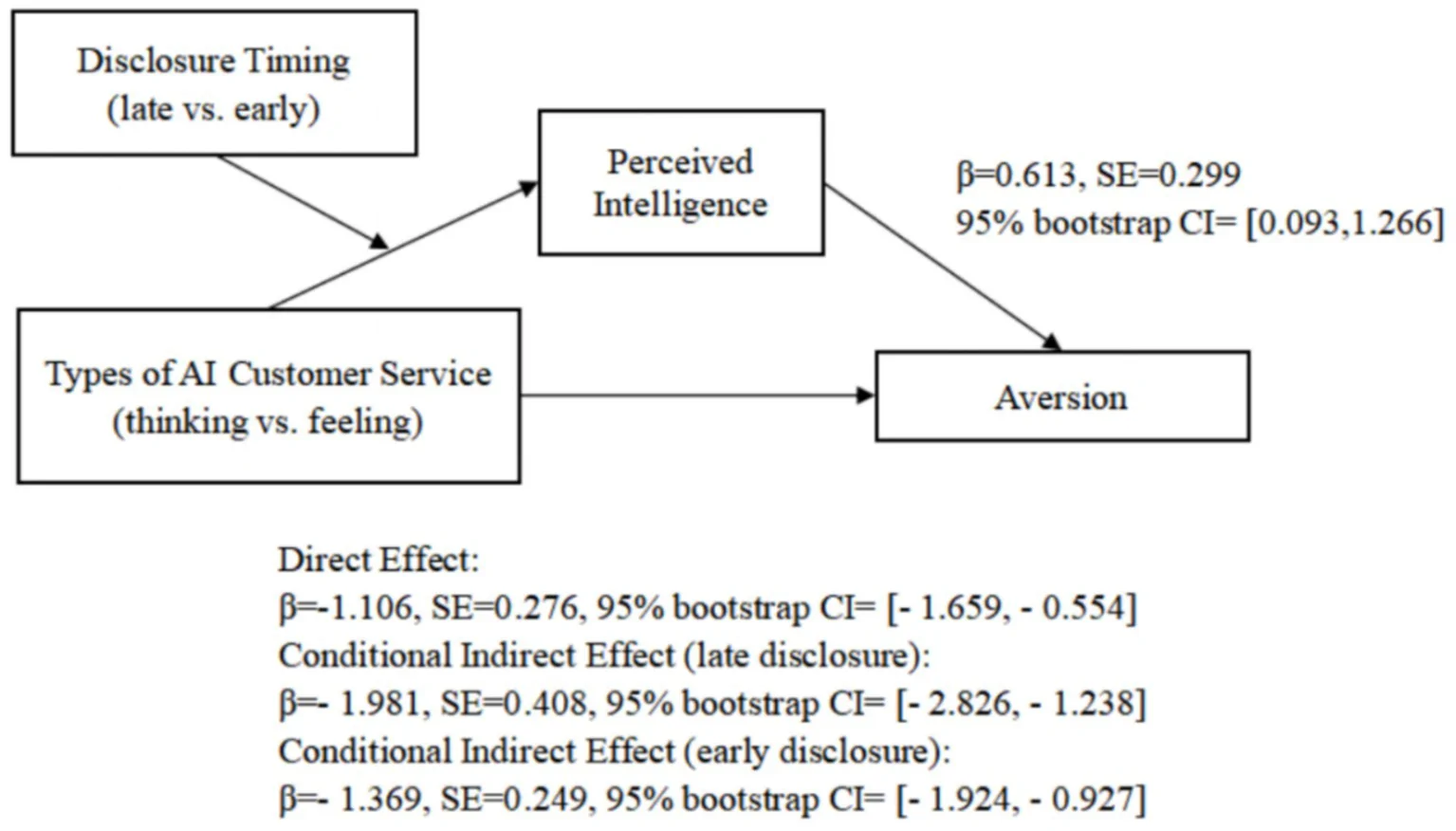
Mediated moderation model (Study 3).

## 8 Discussion

### 8.1 Findings and theoretical implications

This study examined how different forms of AI service role enactment influence user aversion in online educational service encounters. Across three experiments, the findings show that feeling AI generates greater user aversion than thinking AI. Perceived intelligence mediates this effect, such that feeling AI is perceived as less intelligent, which in turn increases user aversion. AI identity disclosure timing moderates the indirect effect through perceived intelligence. These findings suggest that users’ responses to AI customer service depend on how the service role is enacted and evaluated in the service situations examined in this study.

First, this study extends robotic role theory by demonstrating the relevance of role enactment to AI customer service. Robotic role theory emphasizes that service robots perform frontline roles through observable behaviors and that consumer responses depend partly on the suitability of these enacted behaviors for the service interaction (Blaurock et al., 2022; Schepers et al., 2022). Building on this perspective, this study conceptualizes thinking AI and feeling AI as two different orientations of service role enactment rather than as fundamentally different AI systems or service tasks. The findings show that, although the substantive service task and service outcome were held constant, varying the orientation through which the AI agent enacted the service role produced different levels of user aversion. Specifically, thinking AI, which emphasized analytical, informational, and task-oriented behaviors, generated lower user aversion than feeling AI in the educational service situations examined. This finding suggests that the evaluation of AI customer service may depend on how its enacted role corresponds to the requirements of the service interaction, rather than on the presence of emotional or social capabilities alone.

Second, this study identifies perceived intelligence as an important psychological mechanism through which differences in role enactment influence user aversion. Role theory suggests that role performance is evaluated in relation to the behaviors associated with a particular role (Biddle, 1986). Although prior research shows that emotional and social capabilities can enhance user engagement, communication satisfaction, and interaction quality (Song et al., 2022), our findings suggest that users may place greater emphasis on intelligence-related capabilities when evaluating AI service agents in task-oriented encounters. Consistent with prior research indicating that users evaluate AI customer service partly according to its ability to perform required tasks (Gao et al., 2023), we find that affective and relational communication leads to lower perceived intelligence than analytical and task-oriented communication, which in turn increases user aversion. Thus, perceived intelligence provides a psychological link between how an AI agent enacts its service role and how users respond to that role, reflecting users’ judgments of whether the agent possesses the capabilities required for effective role performance.

Third, this study extends research on AI disclosure in customer service interactions by highlighting the role of disclosure timing in the evaluation of AI service role enactment. Prior research has examined whether AI involvement is disclosed (Atefi et al., 2020; Huang and Dootson, 2022), as well as how the content and specificity of disclosure shape consumer responses (Carney et al., 2026; Susanto and Khaq, 2024). Our findings further show that early versus late disclosure of AI identity changes the extent to which thinking and feeling AI differ in perceived intelligence and user aversion. From the perspective of robotic role theory, disclosure provides information about the nature of the actor performing the service role, which may shape how users interpret and evaluate its enacted behaviors. Thus, AI identity disclosure functions as an informational condition under which consumers evaluate different forms of AI service role enactment, extending prior research by emphasizing when AI identity is disclosed.

### 8.2 Managerial implications

The findings provide several implications for organizations deploying AI customer service in online educational service environments. First, firms should align AI service role enactment with the primary requirements of the service task. In the educational service situations examined in this study, thinking AI generated lower user aversion and higher perceived intelligence than feeling AI. Accordingly, when AI customer service is used for consultation, information provision, and task-oriented learning support, firms should prioritize analytical processing, information quality, and problem-solving capabilities rather than assuming that greater emotional expressiveness will necessarily produce more favorable responses.

Second, firms should distinguish between the communication orientation of an AI agent and the substantive service task it performs. The findings do not suggest that affective communication is inherently undesirable. Rather, they indicate that emphasizing emotional and relational communication may generate less favorable responses when the AI agent is evaluated in the educational service situations examined in this study. Managers should therefore consider whether the observable behaviors through which an AI agent enacts its service role are consistent with the requirements of the service interaction.

Third, the findings highlight the importance of timely AI identity disclosure. Our findings show that early disclosure attenuated the negative effect associated with feeling AI. Firms may therefore benefit from clearly communicating an agent’s AI identity at an early stage of the service interaction, particularly when the agent adopts a more affective and relational communication orientation. Such disclosure can help users understand the nature of the service interaction and may reduce unfavorable responses to the AI agent. Firms should maintain accessible human support alongside AI customer service. Early disclosure was accompanied by information about the availability of human customer service and was associated with lower aversion toward feeling AI. Providing users with a clear pathway to human assistance may therefore complement AI-based service, particularly when users require support beyond the AI agent’s capabilities.

### 8.3 Limitations and future research

The experiments used standardized, scripted AI interactions presented through screenshots rather than interactions with a fully autonomous AI system. Although this approach ensured consistency across participants, the limited involvement and realism of the simulated interactions may have influenced participants’ responses. Future research could employ more immersive and interactive designs, systematically examine different service goals or roles across contexts, and use complementary approaches, such as neurophysiological methods, to further explore users’ responses to AI service interactions. Meanwhile, user expectations were not directly measured in the current studies. Future research could examine how prior expectations regarding AI service influence users’ evaluations of different AI service orientations.

## Data Availability

The raw data supporting the conclusions of this article will be made available by the authors, without undue reservation.

## Footnotes

1
www.credamo.com
