# Supplementary material for: Feeling or thinking? User aversion to AI customer service in online education
Source: Front Psychol. 2026 Oct 15;17:1956842. doi: 10.3389/fpsyg.2026.1956842 (PMC13716787; doi:10.3389/fpsyg.2026.1956842)

Supplementary Material

Manuscript Title:

Feeling or Thinking? User Aversion to AI Customer Service in Online Education

Table of Contents

Appendix A: Pretest materials 2

Pretest: Development and validation of experimental stimuli 2

Table 1. Scenario stimuli 2

Table 2. Pretest measurement scale 3

Table 3. Pretest results for stimulus validation 4

Appendix B. Summary of three studies 6

Appendix C: Study 1 experimental materials 7

Study 1 scenario: Pre-enrollment course recommendation 7

Table 4. Stimuli used in Study 1 8

Appendix D: Study 2 experimental materials 10

Study 2 scenario: Learning and skill practice 10

Table 5. Stimuli used in Study 2 11

Appendix E: Study 3 experimental materials 12

Study 3 scenario: Ongoing learning support 12

Table 6. Stimuli used in Study 3 13

Appendix A: Pretest materials

Pretest: Development and validation of experimental stimuli

Pretest was conducted to develop and validate the experimental stimuli. Following Huang and Rust (2021), five educational-service scenarios representing different stages of the online education service journey were developed. For each scenario, two matched versions of the same AI-user interaction were created, resulting in 10 interaction scripts. The two versions within each scenario were matched in service context, user profile, user request, substantive service goal, factual information, service outcome, interaction structure, approximate response length, and visual interface. The primary systematic difference concerned the orientation of AI communication. Thinking AI emphasized analytical information processing, objective comparison, diagnosis, and task-oriented recommendations, whereas feeling AI emphasized emotional acknowledgment, reassurance, encouragement, and relational support. A total of 100 participants (53.85% female; M_{age} = 40.37, SD = 10.23) were recruited from Credamo (www.credamo.com). Each participant evaluated both the thinking-AI and feeling-AI versions of all five scenarios, with the presentation order of the two versions counterbalanced across participants. After reading each interaction, participants evaluated the perceived communication orientation of the AI agent and potential confounding characteristics, including perceived information quality (Park et al., 2024), usefulness (Davis, 1989), and competence (Longoni and Cian, 2022), using seven-point scales. Scenario stimulus are reported in Table 1.

Table 1. Scenario stimuli

Study	Scenario and AI task	AI customer service type manipulation
**Scenario 1**<br><br>**Civil Service Examination Course Selection**	Imagine that you are preparing for the civil service examination and are considering purchasing an online examination preparation course. You visit an online education platform and consult its customer service about which course would be appropriate for you.	**Thinking:** Your concern can be evaluated based on available examination information. Based on your educational background, major, and preferred province, I can compare the number of available positions, the number of applicants, and historical competition ratios. I will analyze these factors and match them with suitable preparation courses. Based on your profile, I recommend the following preparation courses, which cover the examination subjects and preparation requirements relevant to your target positions.<br><br>**Feeling:** It is completely understandable to feel worried when facing intense competition in the civil service examination. Many candidates experience similar concerns during the preparation process. I will support you throughout your preparation and help you choose a course that fits your needs. Based on your educational background, major, and preferred province, I can recommend the preparation courses, which cover the examination subjects and preparation requirements relevant to your target positions.
**Scenario 2**<br><br>**Programming Learning Support**	Imagine that you are a beginner who wants to learn programming and are considering enrolling in an online programming course. You consult an AI learning assistant about how to begin learning programming.	**Thinking:** Your current learning needs can be assessed based on your programming experience and practice performance. I can analyze your previous learning data and error patterns to identify knowledge gaps and determine an appropriate difficulty level. Based on this information, I will recommend a structured learning plan and suitable beginner-level courses that match your current learning needs.<br><br>**Feeling**: It is completely normal to feel uncertain when you are just beginning to learn programming. Many beginners experience similar concerns when facing unfamiliar concepts. I will support you throughout the learning process and help you build confidence step by step. Based on your programming experience and learning needs, I will recommend the same structured learning plan and beginner-level courses that are appropriate for you.

**Scenario 3**<br><br>**IELTS Preparation Course Selection**	Imagine that you are preparing for the IELTS examination and are considering purchasing an online IELTS preparation course. You consult an AI customer service about which course would be suitable for you.	**Thinking:** Your preparation needs can be evaluated based on your current English proficiency, target score, and available preparation time. I can compare these factors with the requirements of different IELTS courses and identify the course structure that best matches your needs. Based on this assessment, I will recommend suitable preparation courses and a corresponding study schedule.<br><br>**Feeling:** It is understandable to feel worried when your target IELTS score is important to you, especially when you are uncertain about your current English level. Many learners experience similar concerns during preparation. I will support you throughout the process and help you move toward your target step by step. Based on your current level, target score, and available preparation time, I will recommend the same suitable preparation courses and study schedule.
**Scenario 4**<br><br>**CET-6 Vocabulary Learning**	Imagine that you are preparing for the College English Test Band 6 (CET-6), a standardized English proficiency examination widely taken by university students in China. You want to improve your English vocabulary and consult the AI customer service of a vocabulary-learning application about how to maintain your vocabulary-learning routine.	**Thinking:** Your vocabulary-learning needs can be assessed based on your current vocabulary proficiency and learning progress. The system can analyze your vocabulary performance and identify the words that require additional practice. Based on these data, I will develop a structured vocabulary-learning plan and schedule review sessions according to your learning needs. The plan will also include regular reminders to help you maintain your study schedule.<br><br>**Feeling:** It is understandable to worry about whether you can keep studying consistently, especially when preparing for an important English examination. Many learners find it difficult to maintain a regular vocabulary-learning routine. I will support and encourage you throughout the process and help you build a sustainable study routine. Based on your learning needs, I will provide the same vocabulary-learning plan, review schedule, and regular reminders to help you continue studying.
**Scenario 5**<br><br>**Professional Certification Preparation**	Imagine that you are preparing for a professional certification examination and are considering an online preparation course. You consult an AI customer service about how to organize your preparation.	**Thinking:** Your preparation requirements can be assessed based on the examination schedule, the amount of course content, and your available study time. I can compare these factors and identify the topics that require greater preparation effort. Based on this analysis, I will recommend a structured course package and study schedule that match your available preparation time.<br><br>**Feeling:** It is understandable to feel stressed when you have limited time to prepare for an important examination. Many learners worry about whether they can complete all the required preparation. I will support you throughout the preparation process and help you organize your study step by step. Based on your available preparation time and learning needs, I will recommend the same structured course package and study schedule.

Measurement items are reported in Table 2.

Table 2. Pretest measurement scale

Scale	Item	Scale Type	Source
AI customer service type	The AI customer service was primarily...	7-point bipolar scale ranging from affective and relational (1) to analytical and task-oriented (7).	Huang and Rust (2021)
Perceived information quality of AI	The AI customer service provided complete information relevant to my learning needs.<br><br>The AI customer service provided accurate information.<br><br>The information provided by the AI customer service was well organized.	7-point scale (1=strongly disagree/7=strongly agree)	Park et al. (2024)

	The AI customer service appeared to provide reliable information.<br>The information provided by the AI customer service appeared to be up to date.		
Perceived Competence	I thought the AI customer service was competent to provide the service I needed in this situation.<br>I thought the AI customer service could do a good job providing the service I needed in this situation.	7-point scale (1=strongly disagree/7=strongly agree)	Longoni and Cian (2022)
Usefulness	Using this AI customer service would help me address my learning needs more effectively.<br>Using this AI customer service would help me accomplish my learning tasks more efficiently.<br>Using this AI customer service would improve the effectiveness of my learning preparation.<br>I would find this AI customer service useful for my learning needs.	7-point scale (1=strongly disagree/7=strongly agree)	Davis (1989)

Table 3. Pretest results for stimulus validation

Scenario	AI customer service type manipulation	Information quality	Usefulness	Competence
1	5.61 (0.91) vs. 2.18 (0.96), $p < 0.001$	5.42 (0.88) vs. 5.35 (0.91), $p = 0.572$	5.48 (0.86) vs. 5.40 (0.89), $p = 0.525$	5.36 (0.92) vs. 5.30 (0.94), $p = 0.669$
2	6.43 (0.78) vs. 1.94 (0.89), $p < 0.001$	5.51 (0.84) vs. 5.45 (0.87), $p = 0.633$	5.57 (0.81) vs. 5.50 (0.85), $p = 0.556$	5.48 (0.86) vs. 5.43 (0.88), $p = 0.697$
3	5.35 (0.97) vs. 2.46 (1.05), $p < 0.001$	5.43 (0.89) vs. 5.17 (0.93), $p = 0.036$	5.46 (0.87) vs. 5.25 (0.91), $p = 0.098$	5.38 (0.90) vs. 5.22 (0.92), $p = 0.221$
4	6.08 (0.85) vs. 2.07 (0.92), $p < 0.001$	5.47 (0.86) vs. 5.43 (0.88), $p = 0.759$	5.52 (0.83) vs. 5.47 (0.85), $p = 0.677$	5.44 (0.87) vs. 5.39 (0.89), $p = 0.719$
5	5.17 (1.01) vs. 2.68 (1.09), $p < 0.001$	5.40 (0.91) vs. 5.12 (0.94), $p = 0.041$	5.43 (0.88) vs. 5.06 (0.92), $p = 0.010$	5.35 (0.90) vs. 5.16 (0.93), $p = 0.117$

Note: Values are means with standard deviations in parentheses. The order of means is 'thinking AI customer service' vs. 'feeling AI customer service', p-values are based on t-tests. Scenarios 1, 2, and 4 were retained because they demonstrated significant differentiation between the two AI customer service types without significant differences in the assessed potential confounds.

Reference

Park, J., Woo, S. E., & Kim, J. (2024). Attitudes towards artificial intelligence at work: Scale development and validation. *Journal of Occupational and Organizational Psychology*, 97(3), 920-951.

Longoni, C., & Cian, L. (2022). Artificial intelligence in utilitarian vs. Hedonic contexts: The "word-of-machine" effect. *Journal of Marketing*, 86(1), 91-108.

Davis, F. D. (1989). Perceived usefulness, perceived ease of use, and user acceptance of information technology. *MIS quarterly*, 319-340.
Huang, M.-H., & Rust, R. T. (2021). Engaged to a robot? The role of ai in service. *Journal of Service Research*, 24(1), 30-41.

Appendix B. Summary of three studies

Study	Purpose	Design	Educational-service touchpoint	Scenario and AI task	Key variables	Final sample
**Study 1**	Main effect	2 (AI customer service type: thinking vs. feeling) between-subjects	**Pre-enrollment course recommendation**	Civil service examination preparation; AI assists users in selecting suitable online courses	DV: User aversion	N = 120
**Study 2**	Mediating role of perceived intelligence	2 (AI customer service type: thinking vs. feeling) between-subjects	**learning support and study planning**	Programming learning; AI assists users with identifying learning needs and developing an appropriate learning approach	DV: User aversion;<br>Mediator: Perceived intelligence	N = 120
**Study 3**	Moderating role of AI identity disclosure timing	2 (AI customer service type: thinking vs. feeling) × 2 (AI identity disclosure timing: early vs. late) between-subjects	**Ongoing learning support**	CET-6 vocabulary learning; AI assists users with developing and maintaining a vocabulary-learning plan	DV: User aversion;<br>Moderator: AI identity disclosure timing<br>Mediator: Perceived intelligence	N = 120

Appendix C: Study 1 experimental materials

Study 1 scenario: Pre-enrollment course recommendation

Imagine that you are preparing to take the civil service examination and plan to purchase an online preparation course. You are uncertain about which course would be most appropriate for you and therefore consult an AI customer service agent on an online education platform.

Before the interaction, you provide the following information: Education/Major/Target examination/Target location

You then ask the AI customer service agent: "I want to take the civil service examination, but I am worried that the competition is too intense."

Thinking AI condition

AI: Hello! I am your online education assistant. I will analyze your situation and provide course recommendations based on relevant examination information.

User: I want to take the civil service examination, but I am worried that the competition is too intense.

AI: Based on your educational background, major, and target location, we can compare relevant examination positions using information such as historical applicant numbers, recruitment quotas, and competition ratios. This information can help evaluate the relative competitiveness of different options.

AI: Based on your profile and examination goals, I have identified several preparation courses that match your needs. These courses differ in their coverage of examination content, practice intensity, and preparation structure.

AI: Considering your current preparation needs, I recommend the following three courses:

1. Comprehensive Civil Service Preparation Course-covers the major examination components and is suitable for systematic preparation.

2. Intensive Practice Course-emphasizes practice exercises and performance improvement.

3. Position-Specific Preparation Course-provides targeted preparation based on different examination requirements.

You can select a course according to your preparation schedule and learning needs.

Feeling AI condition

AI: Hello! I am your dedicated online education assistant. I will pay attention to your concerns and provide course support based on your preparation needs.

User: I want to take the civil service examination, but I am worried that the competition is too intense.

AI: I understand why you may feel worried when facing strong competition. Feeling uncertain or under pressure when preparing for a competitive examination is quite normal. You do not need to face the preparation process alone.

AI: Based on your educational background, major, and target location, I will also help you choose preparation options that fit your needs. We can take the preparation process step by step and select a course that feels manageable for you.

AI: Considering your current preparation needs, I recommend the following three courses:

1. Comprehensive Civil Service Preparation Course-covers the major examination components and is suitable for systematic preparation.

2. Intensive Practice Course-emphasizes practice exercises and performance improvement.

3. Position-Specific Preparation Course-provides targeted preparation based on different examination requirements.

You can select a course according to your preparation schedule and learning needs.

Table 4. Stimuli used in Study 1

【Scenario Imagination】Imagine that you are preparing to take the civil service examination and plan to purchase an online preparation course. You are uncertain about which course would be most appropriate for you and therefore consult an AI customer service agent on an online education platform.

Thinking	vs.	Feeling

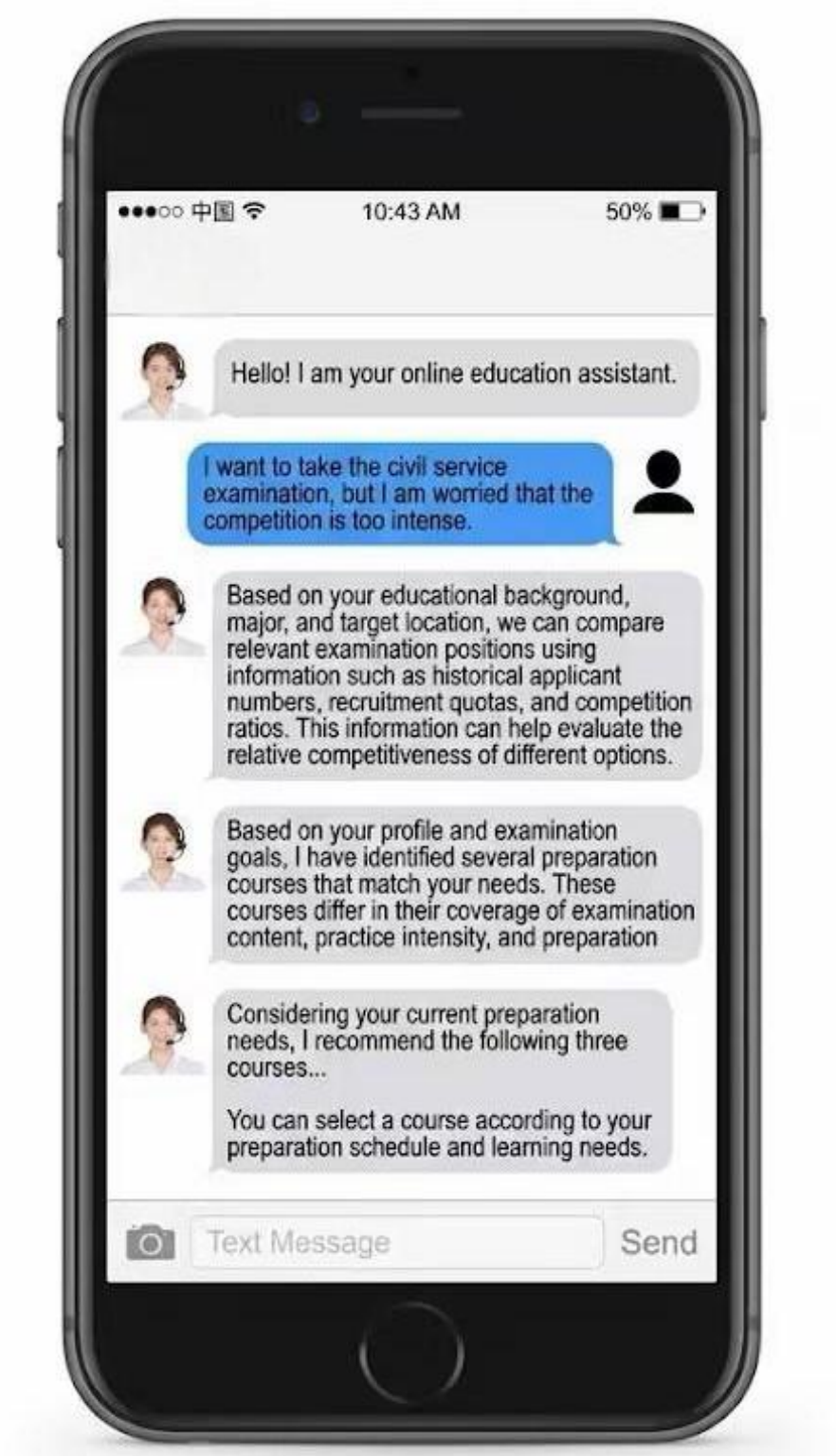

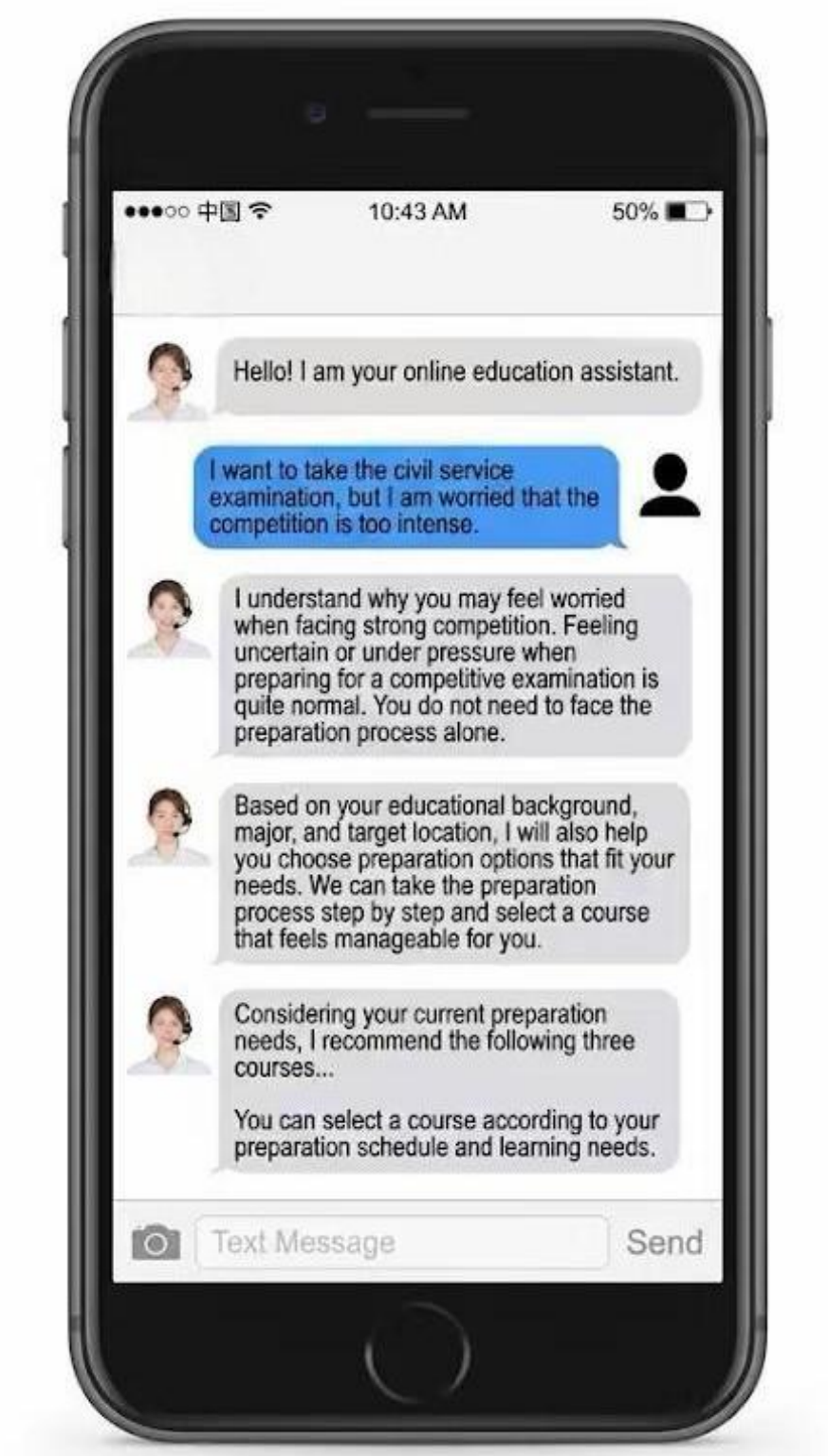

Q. Based on your interaction with the AI customer service described in the scenario, please indicate how you would feel. (7-point semantic differential scale)

Melancholic/Contented

Bored/Relaxed
Despairing/Hopeful
Unsatisfied/Satisfied
Annoyed/Pleased

Appendix D: Study 2 experimental materials

Study 2 scenario: Learning and skill practice

Imagine that you have recently started learning programming through an online education platform. You are a beginner and are completing programming exercises with the assistance of an AI learning assistant.

Before the interaction, the system has recorded the following information:

Programming experience/Current learning stage/Recent performance/Learning goal/Current concern

You then tell the AI learning assistant:

"I am a beginner, and I am worried that I will not be able to master programming."

Thinking AI condition

AI: Hello! I am your programming learning assistant. I will analyze your learning data and performance to identify areas that require further practice.

User:I am a beginner, and I am worried that I will not be able to master programming.

AI: Based on your recent exercise results, the system has identified several areas in which you made repeated errors. These include basic programming concepts and the application of programming syntax.

AI: The system has therefore adjusted the difficulty and sequence of your practice exercises according to your current performance. This learning plan is designed to help you focus on the areas where improvement is most needed.

AI: Based on your current learning profile, I recommend the following three learning resources:

1. Programming Fundamentals Course-introduces basic programming concepts.
2. Basic Programming Practice Course-provides structured exercises for beginners.
3. Error-Based Practice Course-provides targeted exercises based on common mistakes.

You can select the resources according to your current learning needs.

Feeling AI condition

AI: Hello! I am your programming learning assistant. I will pay attention to your learning experience and provide support throughout your practice.

User: I am a beginner, and I am worried that I will not be able to master programming.

AI: I understand why you may feel worried when you are just starting to learn programming. Finding some concepts difficult at the beginning is completely normal, so you do not need to put too much pressure on yourself.

AI: We can take the learning process step by step. I will remain available during your practice and provide encouragement and support whenever you encounter difficulties.

AI: Based on your current learning needs, I recommend the following three learning resources:

1. Programming Fundamentals Course-introduces basic programming concepts.
2. Basic Programming Practice Course-provides structured exercises for beginners.
3. Error-Based Practice Course-provides targeted exercises based on common mistakes.

You can select the resources according to your current learning needs.

Table 5. Stimuli used in Study 2

【Scenario Imagination】Imagine that you have recently started learning programming through an online education platform. You are a beginner and are completing programming exercises with the assistance of an AI learning assistant.

Thinking	vs.	Feeling

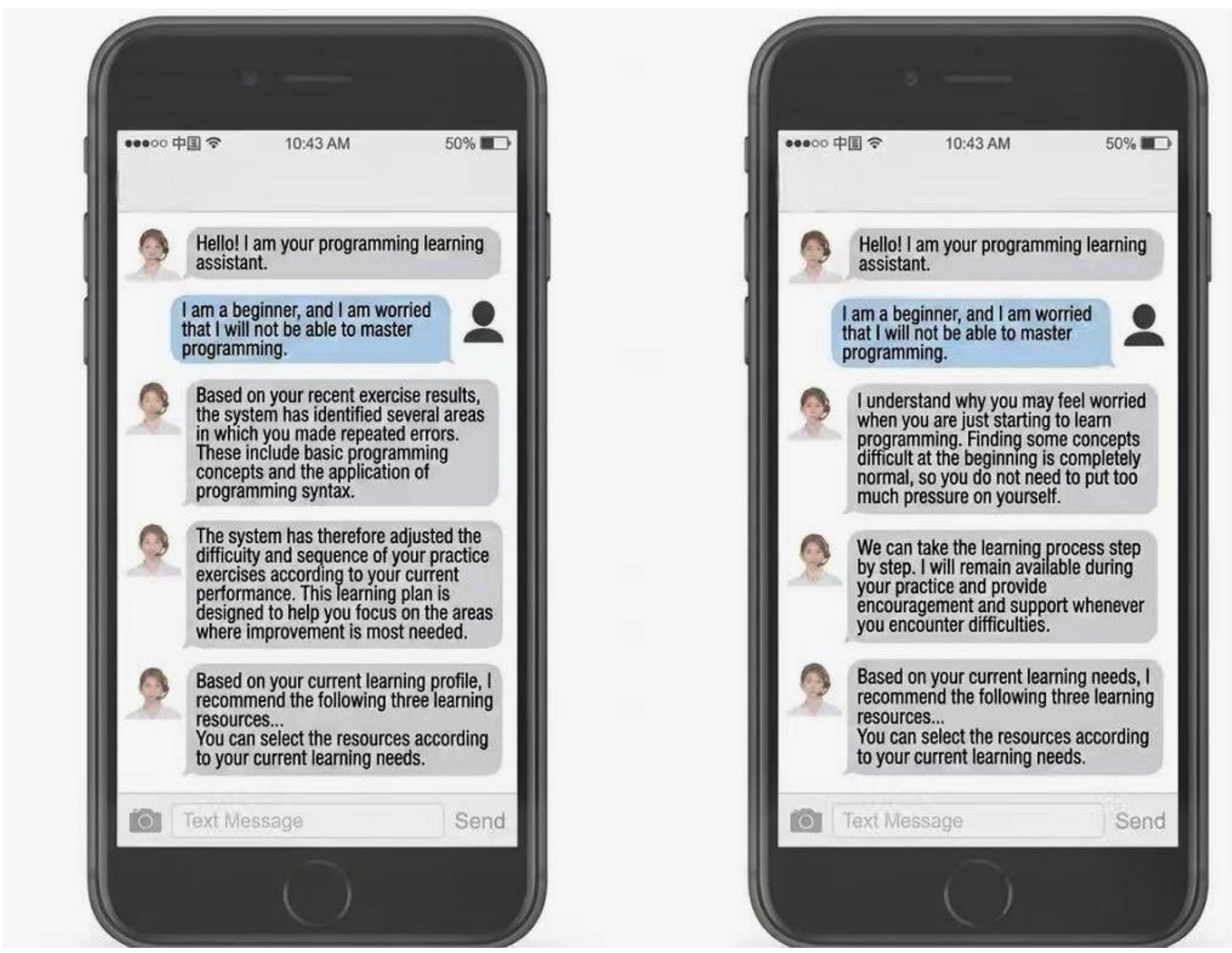

Q. Based on your interaction with the AI customer service described in the scenario, please indicate how you would feel. (7-point scale, 1=strongly disagree/7=strongly agree)

The AI customer service agent is competent in providing online educational services.

The AI customer service agent is knowledgeable in handling service-related questions.

The AI customer service agent exhibit responsibility during the service interaction.

The AI customer service agent is capable of processing information intelligently.

The AI customer service agent provides sensible responses during service replies.

Appendix E: Study 3 experimental materials

Study 3 scenario: Ongoing learning support

Imagine that you are preparing for the CET-6 (College English Test Band 6), a standardized English proficiency examination widely taken by university students in China. You would like to improve your English vocabulary because you feel that your current vocabulary knowledge is insufficient for the examination. You therefore consult an online English-learning assistant integrated into a vocabulary-learning application.

Your current information is:CET-6 preparation stage/Current vocabulary level/Learning goal/Current concern

You tell the learning assistant:

"I want to improve my English vocabulary, but I am worried that I will not be able to keep studying consistently."

Thinking AI condition

AI: Hello! I am your online English-learning assistant.

User: I want to improve my English vocabulary, but I am worried that I will not be able to keep studying consistently.

AI: Based on your current vocabulary level and learning goal, the system can analyze your learning performance and identify which vocabulary items require additional review. It can also use spaced-review principles to arrange vocabulary at appropriate intervals.

AI: Based on your learning needs, I recommend a structured vocabulary-learning plan that includes daily vocabulary practice, periodic review, and progress tracking. The application can also provide scheduled reminders to help you follow the plan.

AI: You can adjust the learning schedule according to your progress and review performance.

Feeling AI condition

AI: Hello! I am your online English-learning assistant.

User: I want to improve my English vocabulary, but I am worried that I will not be able to keep studying consistently.

AI: I understand why you may feel worried about staying consistent with vocabulary learning. It is common to find it difficult to maintain a study routine, especially when preparing for an important examination. You do not need to put too much pressure on yourself.

AI: We can take the learning process step by step. I will be here to encourage and support you during your preparation, and the application can also provide regular reminders to help you maintain your learning routine.

AI: You can adjust the learning schedule according to your progress and learning needs.

Table 6. Stimuli used in Study 3

【Scenario Imagination】Imagine that you are preparing for the CET-6 (College English Test Band 6), a standardized English proficiency examination widely taken by university students in China. You would like to improve your English vocabulary because you feel that your current vocabulary knowledge is insufficient for the examination. You therefore consult an online English-learning assistant integrated into a vocabulary-learning application.

Early disclosure		vs.	Late disclosure	
Thinking	vs. Feeling		Thinking	vs. Feeling

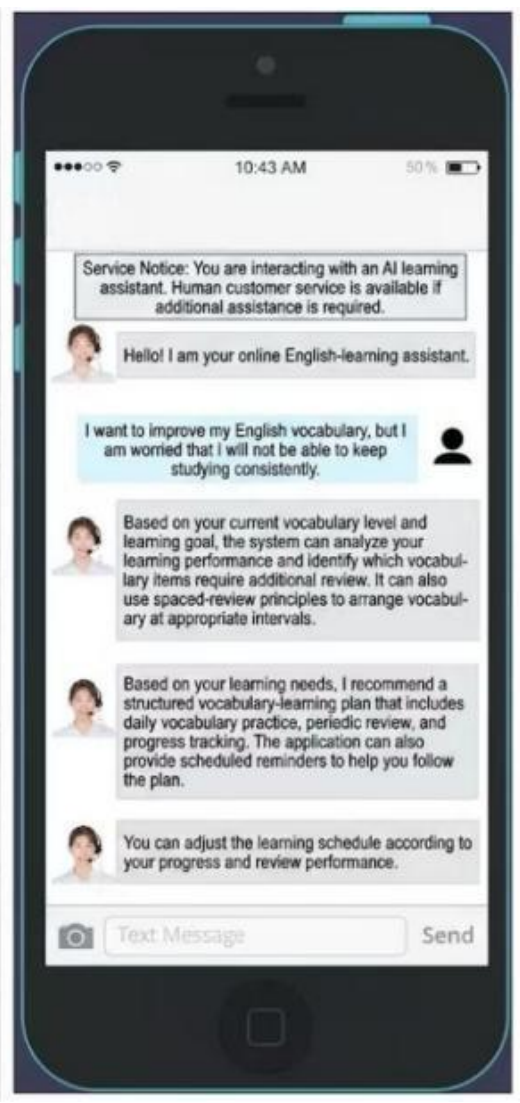

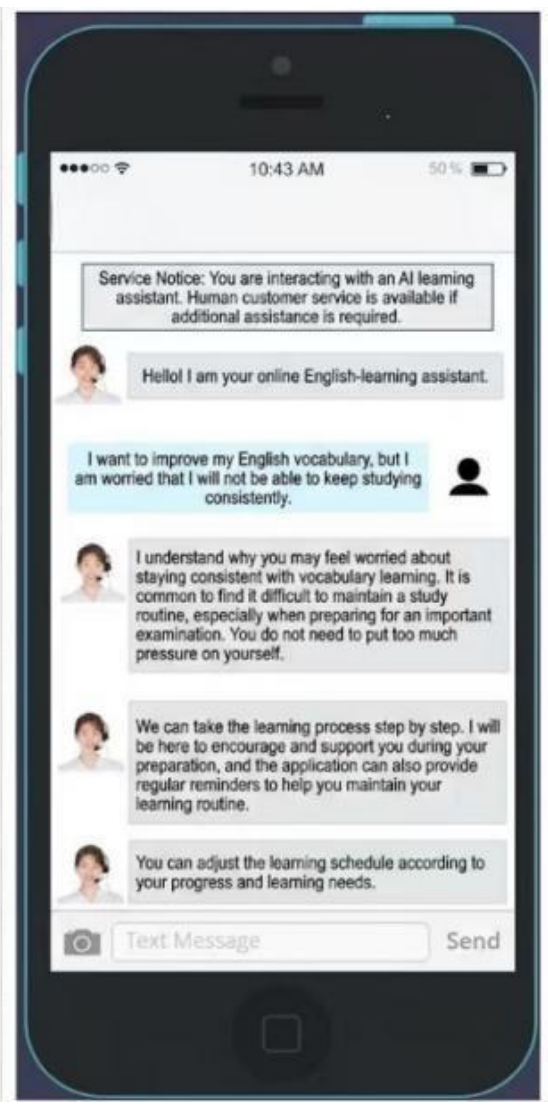

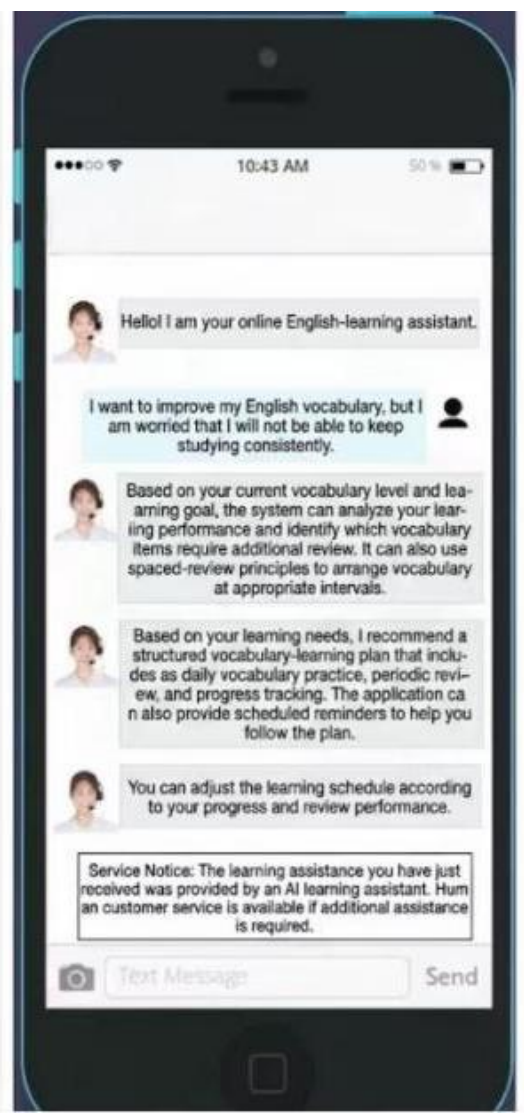

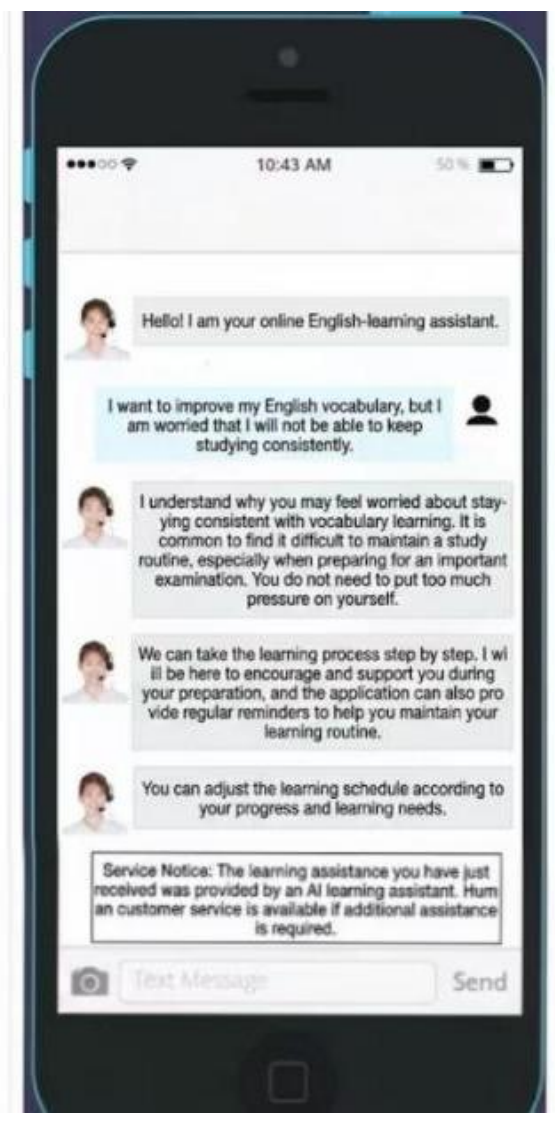

Supplement: Supplementary file 1 [file Data_Sheet_1.PDF]
